# Spinal Cord Infarction with Aortic Dissection

**DOI:** 10.1155/2018/7042829

**Published:** 2018-06-28

**Authors:** Atsuyuki Kawabata, Masaki Tomori, Yoshiyasu Arai

**Affiliations:** ^1^Department of Orthopedic Surgery, Saku General Hospital, 3400-28 Nakagomi, Saku, Nagano 385-0051, Japan; ^2^Department of Orthopedic Surgery, Saiseikai Kawaguchi General Hospital, 5-11-5 Nishikawaguchi, Kawaguchi, Saitama 332-8558, Japan

## Abstract

Spinal cord infarction is an uncommon but devastating disorder caused by various conditions. Aortic dissection is a possible etiological factor and is usually associated with severe chest or back pain. We encountered two cases of spinal cord infarction associated with aortic dissection that presented without typical severe pain, and each case resulted in a different clinical course. Aortic dissection should be considered a cause of spinal cord infarction even if there is little or no pain. The different outcomes in our two patients reflected a difference in their initial functional scores.

## 1. Introduction

Spinal cord infarction is rare, is usually marked by an acute onset, and is associated with substantial motor, sensory, and bladder and bowel dysfunction. The pathologies involved are numerous and include aortic surgery, arteriovenous malformation, aneurysm, and aortic dissection [1]. We encountered two cases of spinal cord infarction with aortic dissection but without the classic symptom of chest or back pain.

## 2. Case Presentation

### 2.1. Case 1

An 85-year-old woman presented to the emergency department with sudden onset of paraparesis, numbness of the legs, and inability to void. She reported having experienced diaphoresis before presentation. She was transferred to our hospital 4 h after onset.

**Figure 1:**
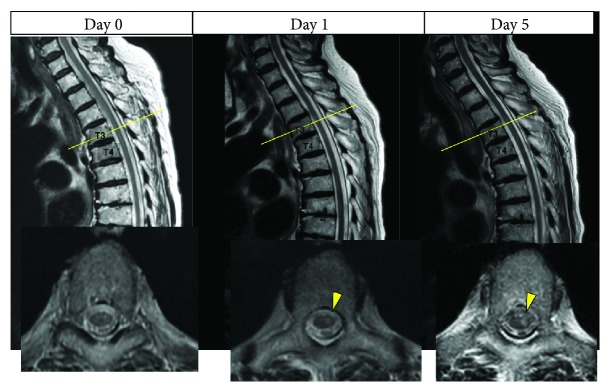
High signal intensity lesion gradually becoming prominent in T2 images.

**Figure 2:**
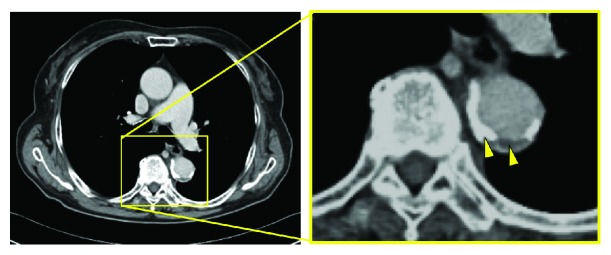
CT showing thrombosed aortic dissection in the thoracic region.

Her medical history was unremarkable apart from hypertension. Her blood pressure was 160/90 mmHg, and her heart rate was regular at 80 bpm. She was alert and oriented but had difficulty standing up. Physical examination revealed dissociated sensory loss below T4 in which sensory perception of vibration and touch was preserved. Muscle function was completely impaired in the left lower extremity globally but somewhat preserved on the right side with a power of 0/3 on the Medical Research Council (MRC) scale. The deep tendon reflex was absent on both sides. Based on these findings, we graded her condition as ASIA grade C. The NIH stroke scale (NIHSS) score was 6 on admission. Six hours after onset, we performed enhanced computed tomography of the whole body and magnetic resonance imaging (MRI) of all spinal lesions. MRI revealed no abnormality, such as ossification, stenosis, a mass, or intramedullary signal changes (Figure 1). CT revealed a thrombosed aortic dissection in the descending aorta (Stanford type B) and severe arteriosclerosis (Figure 2).

**Figure 3:**
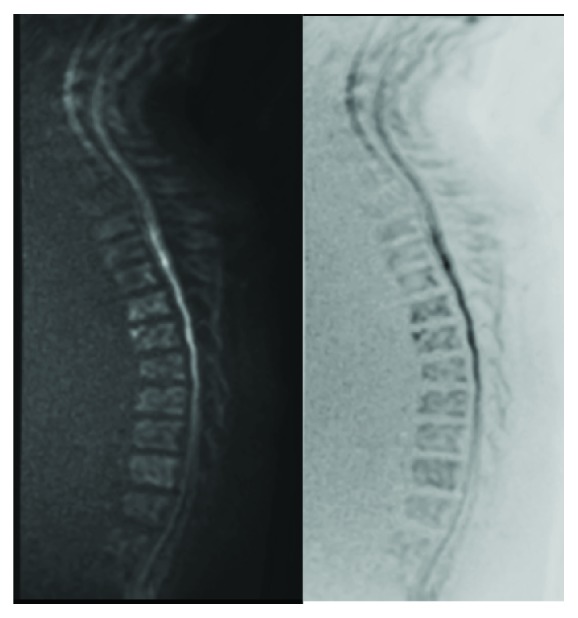
Diffusion-weighted MRI slightly showing abnormality on day 5 (a). Signal changes could be seen a little more clearly on a black and white inverted image (b).

Two days after admission, repeat MRI revealed a linear high signal intensity area on T2-weighted images in the ventral parts of the spinal cord at T3–T10. These areas were confined to the anterior horn in the axial plane. Diffusion-weighted MRI showed slight abnormality on day 5 (Figure 3). Therefore, we made a diagnosis of spinal cord infarction manifesting as sulcal artery syndrome.

Antihypertensive therapy was started. After intensive rehabilitation, her paralysis gradually improved to the point that she was able to walk with the aid of a T-cane and catheter could be removed.

### 2.2. Case 2

The patient was a 68-year-old man who presented to the emergency department after developing sudden complete paraplegia with mild neck pain. He was transferred to our hospital 11 h after onset.

On examination, his blood pressure was 149/74 mmHg and his heart rate was regular at 70 bpm. Complete flaccid paralysis was noted in both lower extremities with a power of 0/0 on the MRC scale as well as loss of all sensation below L1. A digital rectal examination revealed no sensation with absent anal tone. Urinary retention was also present. Based on these findings, we graded his condition as ASIA grade A. The NIHSS score on admission was 10.

**Figure 4:**
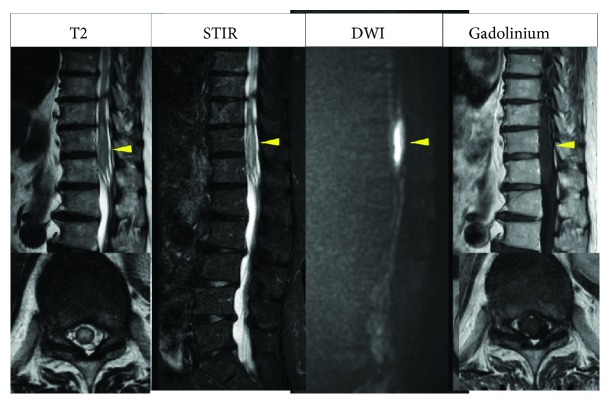
STIR and diffusion-weighted MRI distinctly showing abnormality but no lesion enhancement.

MRI performed 24 h after onset showed high signal intensity in the conus medullaris on T2-weighted images but no compression. Axially, the abnormal signal extended throughout the affected area of the spinal cord. We then performed diffusion MRI, which showed the abnormality more clearly (Figure 4). Spinal fluid was examined, but no abnormality was detected.

**Figure 5:**
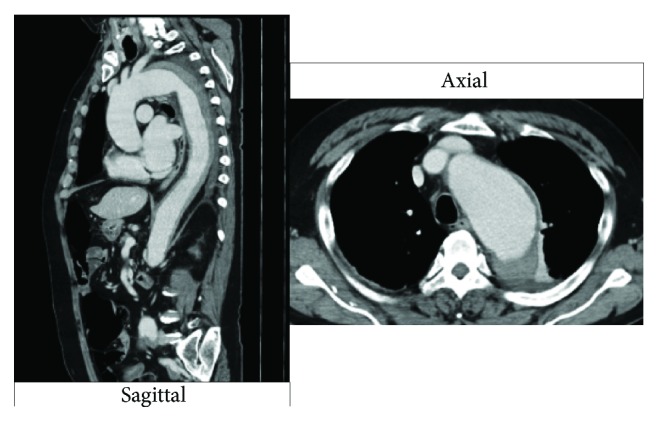
Enhanced CT showing aortic dissection with aortic aneurysm in the descending aorta.

Enhanced computed tomography revealed aortic dissection with an aortic aneurysm in the distal arch. The aneurysm had a diameter of 61 mm, which is an indication for surgery (Figure 5).

Antiedema therapy was started, and rehabilitation was undertaken, during which the patient was monitored carefully. Unfortunately, his physical dysfunction did not improve after 3 months of hospitalization. The patient was finally transferred to another hospital for surgical repair of the aortic aneurysm.

## 3. Discussion

Spinal cord infarction is an uncommon condition that causes sudden paralysis, sensory loss, and urinary and bowel dysfunction. The incidence of spinal cord infarction is 1% that of cerebral infarction [1]. The etiology of spinal cord infarction may be idiopathic (36%), a complication of aortic surgery (25%), a consequence of systemic arteriosclerosis (19%), or attributable to aortic dissection or aortic aneurysm (8%) [2]. Rare causes include cardiac embolism, decompression sickness, coagulopathy, spinal arteriovenous malformation, fibrous cartilaginous embolism, epidural anesthesia, sickle cell disease, vasculitis, and medication [3]. Embolism of the artery of Adamkiewicz is also a possible etiology. It often causes thoracolumbar medullary infarction. Our second case had a thoracolumbar medullary lesion, which possibly arose from embolism of the artery of Adamkiewicz due to dissection of the descending aorta. Regardless of the etiology, aortic aneurysm and aortic dissection are life-threatening conditions and should be investigated carefully in the presence of spinal cord infarction.

Patients with acute aortic dissection often complain of back pain that has a tearing or ripping quality. However, there are some reports of aortic dissection presenting with little or no pain [4, 5]. Classical symptoms of aortic dissection have been reported to be absent in up to 10% of patients [6]. In addition to this, pain could arise from spinal cord infarction alone. Therefore, cases of spinal cord infarction caused by aortic dissection that present without severe pain are quite rare. Although aortic dissection is not a common cause of spinal cord infarction, misdiagnosis can have serious clinical consequences. Even if there is no classic severe pain, enhanced computed tomography should be performed in a patient with acute myelopathy to rule out aortic disease.

The differential diagnosis of spinal cord infarction includes inflammatory disease, autoimmune disease, and vascular disease, such as arteriovenous malformation [7].

One study reported that most of the patients with spinal cord infarction showed no abnormality on MRI [8, 9]. Another study [10] reported that findings on MRI can be normal during the initial few hours to days. However, an abnormality may become apparent on T2-weighted images a week later, so MRI should be repeated within a week. Furthermore, patients with inflammatory or vascular disease have abnormal MRI findings at an early stage. Thus, the absence of abnormal findings on MRI in the initial few hours to days should raise suspicion for spinal cord infarction.

Diffusion MRI has been reported to be more sensitive for detection of spinal cord infarction [8, 9], which was the case in our second patient. A further report suggested that adjacent vertebral infarction confirms spinal cord infarction [11]. However, despite its high specificity, concomitant vertebral infarction has been reported to have low sensitivity for spinal cord infarction. Vertebral infarction was not detected in either of our patients at any stage.

There are no clear guidelines for the treatment of spinal cord infarction [12]. The choices are an antihypertensive, antiplatelet, or anticoagulant agent, a corticosteroid, or continuous spinal drainage, depending on the cause. Anticoagulant agents should be used cautiously in patients with aortic dissection.

Although the prognosis of spinal cord infarction has been reported to be more benign than that of cerebral stroke in terms of cognitive function and mental state, about half of patients with spinal cord infarction require a wheelchair or bladder catheterization [13, 14].

One of our patients had a benign course and was able to walk using a T-cane at the time of discharge from the hospital. In contrast, our other patients had no functional recovery after 3 months of hospitalization. Given a report suggesting that patients with ASIA scores of A or B have worse outcomes than other scores [14], it is possible that the different outcomes in our two patients reflect a difference in their initial functional score.

In summary, we encountered two cases of spinal cord infarction associated with aortic dissection that presented without typical severe chest or back pain. Aortic dissection should be considered a cause of spinal cord infarction even if there is no or little pain.
